# Age Determination and Growth Characteristics of the *Potentilla griffithii*: A Comparison of Two Different Habitats in Western Sichuan Plateau, China

**DOI:** 10.3390/plants12162920

**Published:** 2023-08-11

**Authors:** Xiulong Zhang, Xingxing Lin, Dandan Wei, Weikai Bao, Bin Hu

**Affiliations:** 1 CAS Key Laboratory of Mountain Ecological Restoration and Bioresource Utilization & Ecological Restoration and Biodiversity Conservation Key Laboratory of Sichuan Province, Chengdu Institute of Biology, Chinese Academy of Sciences, Chengdu 610041, China; zhangxl0507@outlook.com (X.Z.); linxingxing21@mails.ucas.ac.cn (X.L.); weidd@cib.ac.cn (D.W.); hubin@cib.ac.cn (B.H.); 2 University of Chinese Academy of Sciences, Beijing 100049, China

**Keywords:** age determination, perennial herbaceous, alpine habitat, adaptation strategy

## Abstract

This study proposes a rapid and non-destructive technique for determining the age of *Potentilla griffithii* individuals in the field by observing the sequence of leaf scars. Based on two- to three-year-old *P. griffithii* seedlings, planted in a common garden in the western Sichuan Plateau, China, the study found that the rates of basal leaf production were consistent, with leaves growing from March to April and falling off from October to December, leaving behind basal leaf scars. Thus, the age of individuals in situ could be determined by counting the leaf scars. Through this method, we determined the age structure and growth strategy of *P. griffithii* populations in two typical habitats in the western Sichuan Plateau. In open land habitats, the age structure of *P. griffithii* populations was relatively younger compared to understory habitats. In open land, *P. griffithii* tends to allocate more photosynthate terminal organs (leaves and fine roots) to absorbing more resources, as well as to its reproductive organs (flower stems and aggregate fruits), to expand the population. The *P. griffithii* population in the understory habitat is in its middle-age stage and concentrates more photosynthate in the coarse root part (e.g., the high coarse root mass fraction (FRMF)) to support the plant. Additionally, we found a significant correlation between *P. griffithii* plant age and various traits in open land habitats. Therefore, we conclude that plant age can be used as a good predictor of plant growth condition in open land. These results allow for predicting ecological processes, based on the ages and traits of *P. griffithii* plants, providing a theoretical basis to support the large-scale breeding of *P. griffithii*.

## 1. Introduction

The genus Potentilla belongs to the Rosaceae family; it is primarily distributed in the temperate, arctic, and Alpine zones of the northern hemisphere [1]. *Potentilla griffithii*, a widely distributed perennial stoloniferous herbaceous plant found in western Sichuan Plateau alpine meadows, exhibits strong stress resistance and wide ecological adaptability. It can adapt well to the complex and changeable alpine habitat and is an important germplasm resource for the ecological restoration of degraded alpine meadows. Current research on *P. griffithii* found that *P. griffithii* is a potential hyperaccumulator with strong tolerance and enrichment ability in terms of Zn, Pb, and Cd, providing a new option for the phytoremediation of soil contaminated by heavy metals [2,3]. However, our understanding of biomass allocation, morphological characteristics, and age structure in different habitat populations of *P. griffithii* is still insufficient, particularly in ecologically fragile regions. This limitation hinders breakthroughs in the key technology of the large-scale domestication of potentillas.

Plant age is an essential parameter for interpreting plant adaptation strategies over time. For many years, plant age has been considered to affect many critical ecological processes. Furthermore, age structure plays a central role in understanding population dynamics and the life history of plants in certain habitats [4,5,6]. Throughout the life history of plants, they undergo variable physiological changes [7,8,9,10,11], biomass allocation changes [12,13], morphological changes [14,15,16,17], and changes regarding reproduction [18,19,20,21], which ultimately influence their growth strategies. Therefore, numerous studies have long been interested in determining the age of individual plants and making efforts to clarify the relations between plant age and plant growth strategies [22,23,24,25]. However, to date, there is still a large number of species where their ages cannot be accurately determined, due primarily to current technical constraints, such as with the perennial grasses, which limits the study of age characteristics and our understanding of the relationship between plant age and growth.

The most common method for determining plant age is by using annual growth rings, especially for woody species in temperate zones [26,27,28]. In recent years, this method has also been widely applied to other regions and more species, such as tropical tree species [29,30,31] and herbaceous plants in Europe and North America [32,33,34]. However, for many perennial grass species, although numerous studies have shown that the annual ring structure can be clearly identified in the xylem of the main root [35,36], there are still a considerable number of perennial grasses that only have vascular tissue and, thus, cannot produce an obvious growth ring, which limits the determination of age characteristics. Currently, there is no efficient and rapid method for estimating plant age that can be universally applied to perennial grasses. Thus, for such species, it is necessary to find a method to accurately characterize their age. Apart from the annual ring-growth method, plant age can also be estimated based on plant growth characteristics. Previous studies linking plant morphological development, plant biomass accumulation, and allocation to plant age [13,17,37] utilized time-consuming measurements over long periods of time or were dependent upon questionable assumptions. Additionally, these studies rely more on model fitting, with varying degrees of complexity; the parameters are largely affected by the environment, lacking direct evidence. The “trace tracking method” is an alternative method that has long been applied for determining plant age, such as using leaf scars to age *Rhizophora* seedlings or counting bud scars to determine the age of *Quercus stellata* and *Q. marilandica* [38,39]. However, this approach has not been widely tested for use in perennial plants.

Studies have demonstrated that plant growth strategies are affected by the environment. For instance, there are significant differences in biomass accumulation, allocation, and morphological variation between open land and understory habitats [40]. When comparing similarly aged plants growing under different growth conditions, plants developing under light constraints tend to be smaller at a given age than plants growing under more favorable conditions, as seen by variations in root: shoot ratios [12]. In addition, plants grown in low light conditions (e.g., high canopy openness) frequently show increased allocation to shoots, while plants with abundant light resources show increased allocation to roots, in order to attain functional equilibrium based on their most limiting resources [41,42]. Consequently, corresponding changes occur in plant morphology and quantitative parameters (e.g., leaf number, flower stem number, and fruit number). Significant effort has been dedicated to understanding how environmental factors drive this variation in plant strategies [43,44]. However, plant age, which is believed to be an important source of variations in functional traits, has received relatively less research attention than variations due to environmentally driven plant growth strategies [45,46,47]. As plants age, the relative importance of resource availability and abiotic circumstances shifts, resulting in changes in plant strategy. These modifications can be seen in biomass allocation patterns and stress tolerance methods [48,49] and responses to environmental changes [50,51,52]. These shifts in strategies along with plant age are important for predicting how plants respond to changing environments, as individuals may exhibit varying degrees of sensitivity to certain stresses at different stages of development.

According to the leaf economics spectrum [53], plants tend to shift from ‘‘faster’’ to ‘‘slower’’ growth and nutrient-use strategies with plant age [54,55]. Additionally, plants tend to invest a higher proportion of resources in stems compared to leaves over time [56]. However, previous studies on the variation of functional traits with plant age have measured only a few developmental stages and have utilized cross-sectional approaches instead of conducting longitudinal studies (i.e., tracking individuals over time). These limitations make it difficult to determine whether an observed trait variation is attributable to the environment or to plant development [46,50,51,54,57,58].

Previous studies have found that the basal leaves of potentillas have obvious interannual growth patterns [1]. According to the above background, we thus hypothesize that the age of *P. griffithii* can be determined by leaf scars. Therefore, this study focuses on *P. griffithii*, which is native to two different habitats in the western Sichuan Plateau, China, to explore age-related structure changes and variations in age–trait relationships. Specifically, we aim to answer the following questions:

(1) Can the age of *P. griffithii* plants be determined by leaf scars? If yes, what is the age structure character of *P. griffithii* in two typical habitats in the western Sichuan Plateau?
(2) What survival strategies does *P. griffithii* adopt in different habitats in the western Sichuan Plateau?
(3) Are age–trait relationships consistent enough to allow a general model to accurately predict plant growth conditions, based on plant age?

To address these questions, we first planted *P. griffithii* in a common garden to confirm research question (1). We then used the measurements of plant traits and plant age in two natural habitats to answer research questions (2) and (3).

## 2. Results

### 2.1. Age Determination of P. griffithii in Common Garden Individuals

After three growing seasons from 2021 to 2023 for the observation of homogeneous garden plants of *P. griffithii*, we found that the new leaves of *P. griffithii* emerged from March to April and started to wither in October. The growth mode of the basal leaves was whorled, and only one layer was grown each year.

### 2.2. Age Distribution

**Figure 1:**
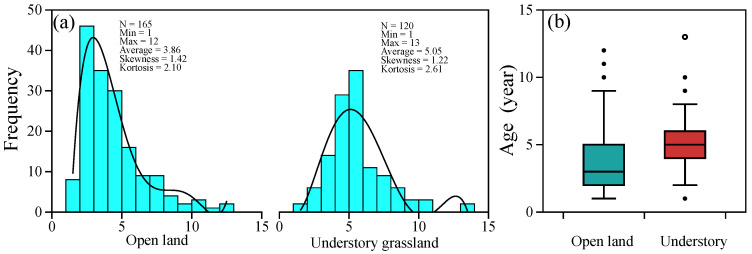
Age distribution characters of *Potentilla griffithii* individuals in two typical natural habitats (**a**), and the boxplot of age data in open land and understory (**b**).

Based on the observation of *P. griffithii* in the common garden environment, we determined the age of each individual. The range of the age distribution between the two sites did not vary considerably; the maximum age was 12 in open land and 13 in the understory (Figure 1a). The mean age of the open-land individuals is 3.86, which is younger than that of 5.05 in forest conditions (Figure 1).

### 2.3. Trait Comparison

The biomass, the plant organ mass fraction, and the whole-plant morphological traits of *P. griffithii* individuals in sites with different light conditions are listed in Table 1. The plant organ biomass and their allocation significantly differed between the sites. The above-ground dry mass, above-ground mass fraction, leaf mass fraction, and fine root mass fraction in open land were significantly higher than those in plants from understory grassland (Table 1). The below-ground dry mass, root-shoot ratio, below-ground mass fraction, and coarse root mass fraction were lower in open-land individuals than in understory individuals. As for total plant dry mass and stem dry mass fraction, no difference was observed between the two sites.

The whole-plant morphological traits, except for total leaf area, stem diameter, total root length, and specific root length, varied significantly between the two habitats (Table 1). Total leaf number, stem number, and leaf mass per area were lower in open-land individuals, whereas the total stem length and root diameter were higher in open-land individuals (Table 1).

### 2.4. Relationships between Age and Plant Traits

**Figure 2:**
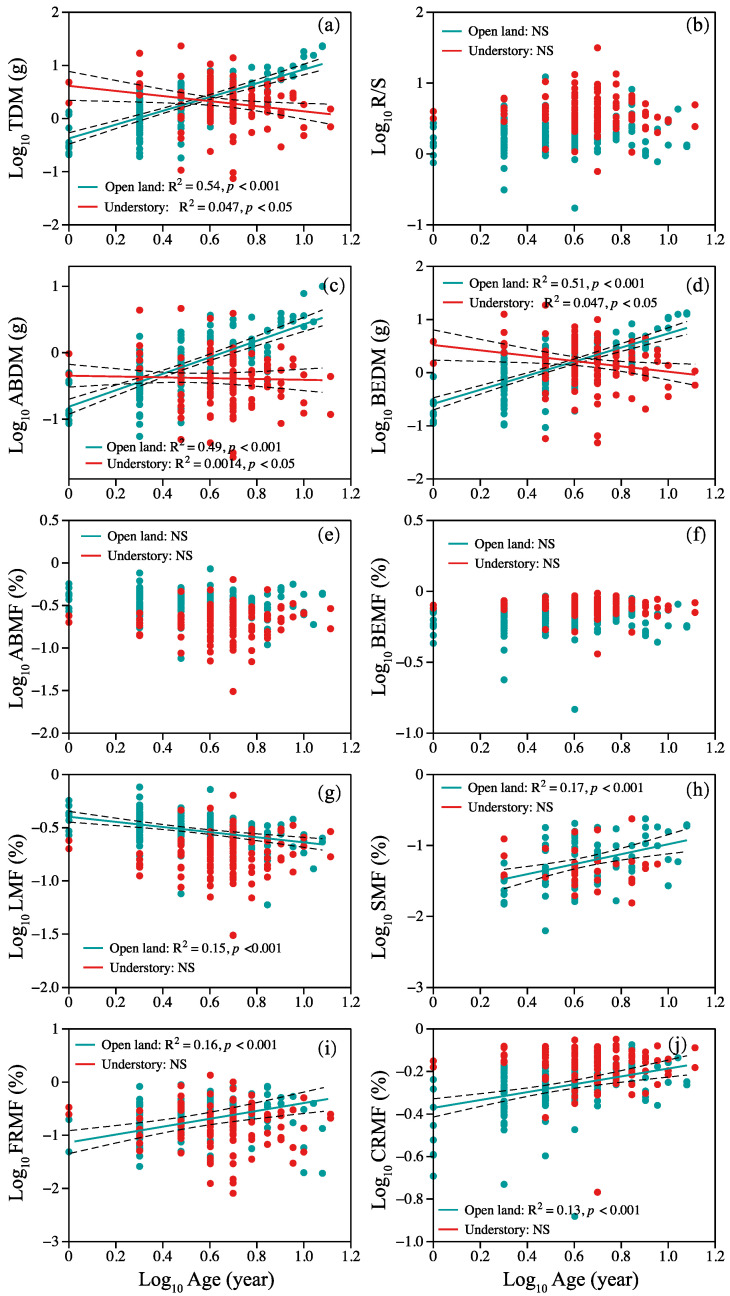
Relationship between plant age and (**a**) TDM, (**b**) R/S ratio, (**c**) ABDM, (**d**) BEDM, (**e**) ABMF, (**f**) BEMF, (**g**) LMF, (**h**) SMF, (**i**) FRMF, and (**j**) CRMF. Symbols: open land (blue circles), understory grassland (red circles). Significance of the regression lines: Statistically significant. Dotted lines: 95% confidence interval. A summary of the regression statistics is shown in Table S1 in the Supplementary Materials. The full names of plant traits can be found in Table 2.

Correlations between plant age and plant organ biomass and allocation are shown in Figure 2. Overall, two different patterns were observed. In open-land individuals, a positive linear correlation between TDM, ABDM, BEDM, SMF, CRMF, and age was observed (Figure 2a,c,d,h,j), but the opposite trend between TDM, ABDM, BEDM, and age was shown in understory individuals (Figure 2a,c,d). Leaf mass fraction decreased linearly with age in open-land individuals (Figure 2g). Fine root mass fraction and age showed a nonlinear pattern, and statistically significant second-order terms were present (Figure 2i). We found no significant relationship between R/S, ABMF, BEMF, and age in plants from the two sites (Figure 2b,e,f).

**Figure 3:**
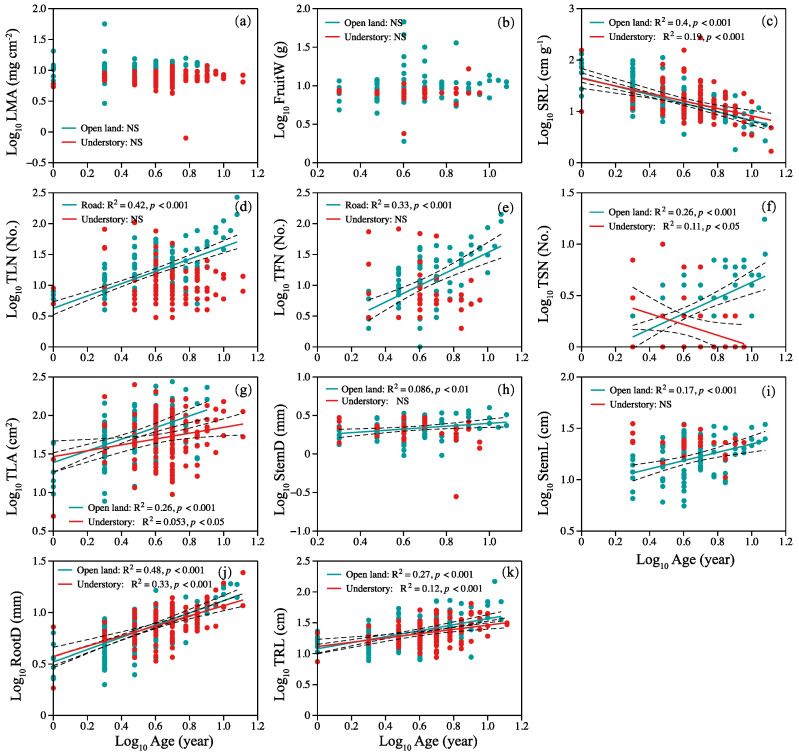
Relationship between plant age and (**a**) LMA, (**b**) FruitW, (**c**) SRL, (**d**) TLN, (**e**) TFN, (**f**) TSN, (**g**) TLA, (**h**) StemD, (**i**) StemL, (**j**) RootD, and (**k**) TRL. Symbols: open land (blue circles), understory grassland (red circles). Significance of the regression lines: Statistically significant. Dotted lines: 95% confidence interval. A summary of the regression statistics is shown in Table S1 in the Supplementary Materials. The full names of plant traits can be found in Table 2.

In open-land individuals, specific root length decreased linearly with plant age (Figure 3c), but TLN, total fruits number, TSN, TLA, StemD, StemL, RootD, and TRL increased with plant age (Figure 3d–k). In the habitat of the understory, SRL and total stem number decreased linearly with plant age (Figure 3c,f). Total leaf area, root diameter, and length increased with plant age (Figure 3g,j,k). There was no significant relationship between LMA, mean fruit weight, and plant age (Figure 3a,b).

**Figure 4:**
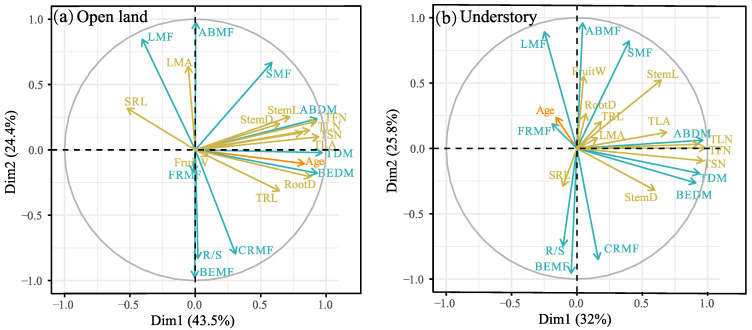
Principal component analysis (PCA) biplot showing the relationship between plant traits and plant age. (**a**) Open land, (**b**) understory grassland. The plant traits of biomass and allocation are indicated by blue arrows pointing in the direction of increasing values. Plant morphological traits are indicated by yellow arrows pointing in the direction of increasing values. Full names corresponding to the abbreviated trait names in the figures are given in Table 2. Detailed PCA results refer to Table S2 in the Supplementary Materials.

The principal component (PCA) analysis results are shown in Figure 4 and Table S2 in the Supplementary Materials. In open-land individuals, the first PCA axis showed strong loadings regarding plant age, BEDM, ABDM, TDM, SRL, TLN, TFN, TSN, TLA, TRL, RootD, StemD, and StemL, and accounted for 43.5% of the total variation. The second axis had strong loadings on CRMF, BEMF, LMA, SMF, ABMF, R/S, and LMA (accounting for 24.4% of the total variation) (Figure 4a). In the understory individuals, the first PCA axis showed strong loadings on BEDM, ABDM, TDM, TLF, TFN, TSN, TLA, StemD, and StemL, and accounted for 32% of the total variation. The second axis had strong loadings on CRMF, BEMF, LMA, SMF, ABMF, R/S, and LMA (accounting for 25.8% of the total variation) (Figure 4b).

## 3. Discussion

### 3.1. Leaf Scars Can Be Used for Aging P. griffithii

To verify the hypothesis and resolve research question 1, we planted a large number of *P. griffithii* seedings to observe the plant’s growth characteristics. After three years of observation, we found that the leaves of *P. griffithii* started to fall off in the period from October to December, leaving a leaf scar. This phenomenon indicates that *P. griffithii* demonstrates constant annual leaf scar production. Therefore, we believe that leaf scars can be used as a basis for determining the age of *P. griffithii* plants in the field. Our result provided a more robust means to age the seedings of *P. griffithii.* This characteristic of seeding growth offers a rapid and simple means by which to age *P. griffithii* individuals and quickly characterize the demographic patterns in *P. griffithii* communities in the field.

Age structure is considered to reflect the problems faced by plant populations and is used to help predict the direction of population regeneration [59]. Based on the above leaf-scar tracking method, we determined the age structure of *P. griffithii* in two typical habitats in the western Sichuan Plateau. According to age distribution, we found that the age structure of *P. griffithii* in the open land environment was left-leaning (Figure 1), which implies that the highest survival rate of the population is in open land. This may mainly be due to the good light conditions in the open land, as available light plays a vital role in the germination, growth, and survival of plant seedlings in light-demanding species [60,61]. However, the age structure of plants in understory grassland shows a normal distribution; that is, the number of young and old individuals is small, but the number of middle-aged individuals is large, indicating the low survival rate of subsequent generations, constituting an unfavorable situation for the plant population’s continuation and development. In addition, the proportion of older-age specimens (>9 years old) in open land is greater than that in specimens from the understory grassland (Figure 1), which finding was consistent with previous studies that *P. griffithii* plants always extend their lifespan when coping with harsh environments [62].

### 3.2. Growth Strategies of P. griffithii in Two Habitats

The variations in plant growth and biomass allocation reflect the adaptation of plants to the environment and can, thus, provide a reference for the long-term adaptation of plant individuals and populations to environmental change [56]. Our results revealed a significant difference in plant growth, biomass accumulation, and allocation of *P. griffithii* between the two sites (Table 1). We observed significantly higher values of ABDM, ABMF, LMF, SMF, FRMF, TLN, TSN, TFN, TLA, FruitW, and LMA in open land (Table 1). Increased leaf fraction, fine root fraction, leaf area, and number (higher LMF, FRMF, TLA, and TLN) allow for the better exploitation of light, water, and nutrients [63]. Higher levels of SMF, TSN, and TFN indicate the high reproductive capacity of *P. griffithii* in open land. Although a higher LMA limits plant photosynthesis, this limitation is complemented by leaf numbers. Higher LMA, on the other hand, can protect leaves from damage due to strong light [64].

In addition, our results revealed lower R/S ratios in open-land individuals (Table 1). In general, in order to limit plant growth to an equal extent (according to the optimal growth theory), plants will balance the allocation of photosynthate to their above- and below-ground parts [65,66]. Multiple environmental factors are driving the changes in R/S, such as variations in nutrient availability and above-ground stress [56,67]. We considered that one critical factor is light intensity in the present study, which is considered as a primary factor limiting plant growth in alpine ecosystems [62]. The decrease in light intensity from open land to understory grassland is due to the gradually closing canopy structure [40]. The good light conditions in open land increase the above-ground proportion and further improve the additional light uptake capacity. These strategies corresponded to the characteristics of a young age-structure population.

### 3.3. Do Age–Trait Relationships Allow a General Model to Accurately Predict Plant Growth Conditions from Plant Age?

We are uncertain how best to answer this question, as the correlations between plant age and plant traits are evidently constrained by growth environments. Our results showed a strong correlation between plant age and traits in open land. However, at the understory grassland site, plant age was not a good predictor (Figure 2, Figure 3 and Figure 4). These findings suggest that the plant growth conditions of *P. griffithii* could be predicted well by plant age in an open-land environment. Specifically, total above- and below-ground biomass accumulation increased with plant age, but the root: shoot ratio did not change with plant age. This implies a balanced-growth strategy throughout the whole life history of *P. griffithii* [12]. However, there exists a trade-off between leaves and flower stems in the above-ground part, and a fine–coarse root trade-off in the below-ground part. Generally, with an increase in plant age, more biomass tended to be allocated to stems and coarse roots, which indicates that the reproductive capacity increased with plant age, and more coarse roots also contribute to the extension of its lifespan [56,68]. At the understory grassland site, we found that the total biomass of above- and below-ground biomass showed a consistent decrease with plant age, along with no significant change in the mass fraction of each organ (Figure 2). This first implies that there are resource limitations in plant growth along with plant age development, as plants growing under resource-limited conditions will generally be smaller than those in more compatible conditions at a given age. Second, the constant R/S and other organ mass fraction reveal the plant’s balanced-growth strategies throughout its life cycle [12].

In addition to biomass and its allocation, significant differences in the correlations between plant age and plant morphological traits are also observed between the two sites. Plant organ numbers, total leaf area, and flower stem (diameter and length) in open land increased with plant age, implying its high capacity for resource capture (e.g., light), and reproductivity with the increase in plant age. However, in the understory habitat site, the reproductive capacity decreases with increasing plant age (Figure 3). The root morphology (SRL, diameter, and length) showed a consistent trend with plant age in the two sites, indicating consistency in the underground resource acquisition strategy. In addition, the PCA analysis also reveals that plant age could well predict plant growth conditions in open land (Figure 4).

### 3.4. Future Implications

In the present study, *P. griffithii,* seen as a typical perennial grass species native to the western Sichuan Plateau, has great potential application value and enjoys huge market demand in the future ecological restoration program. The results of our study confirm the importance of leaf scars as a potentially useful tool to determine the age of perennial herbs. Additionally, it helps us to better understand the intricate dynamics of *P. griffithii* populations in the wild and their adaptations to climate change. The two habitats that we selected correspond to the early and late stages of the ecological restoration project. The results provide a method by which to predict the effect of ecological restoration using the plant age of *P. griffithii*. In addition, the results of this study can provide a scientific basis for the large-scale breeding of *P. griffithii*.

## 4. Materials and Methods

### 4.1. Plant Species

*Potentilla griffithii* Hook. f. is a perennial grass mainly distributed in Yunnan, Tibet, Guizhou, and Sichuan in China, at altitudes ranging from 2000 m to 3800 m. It generally grows on bare land, hillside grassland, the forest edge, and the understory and has not been artificially introduced or cultivated artificially. To date, the limited studies focused on this species have mainly concentrated on its heavy metal accumulation ability [2,3,69,70]. Its role in ecological restoration has received little research attention.

### 4.2. Environmental Design

The present study involves a two-part experimental process, comprising the common garden experiment and the field experiment. The detailed experimental process is as follows; the landscapes of the two experiment sites are shown in Figure S1 in the Supplementary Materials.

#### 4.2.1. Common Garden Experiment

To explore an efficient method for determining the age of *P. griffithii* individuals in their natural habitats, a seeding experiment was conducted in May 2021 in Xinduqia, Kangding, Sichuan, China (101°36′38″ E, 30°4′45″ N), at an altitude of 3555 m above sea level (m.a.s.l). The mean annual temperature in this region is 1.80 °C, and the average annual precipitation is 528 mm.

Seeds from the *P. griffithii* species plants were collected from their natural habitats in the western Sichuan Plateau (altitude range of 3600–4000 m) in the fall of 2020. Seeds were first air-dried for 10–15 days and then stored at room temperature (−2–10 °C) until sowing. Before sowing, all the seeds were disinfected for 30 min using 2.5% NaClO. Seeds were planted at a depth of 0.5–1 cm in a regularly spaced pattern in May 2021. The planting density was 9.58 g m^−2^, and a total of 626 m^2^ was planted. Weekly watering was performed after planting to prevent early seedling losses. Minimal additional interference was carried out to ensure natural growth conditions.

The seeds produced true leaves about one month after sowing. We obtained annual, biennial, and triennial plants from our experimental site in October 2021, October 2022, and June 2023, respectively. This observation period lasted for three growing seasons, in order to determine the age-related growth characteristics.

#### 4.2.2. Field Experiment

In September 2021, we selected two typical habitats of *P. griffithii* in the Gaoersi Mount area (101°25′40″ E, 30°3′29″ N) of the western Sichuan Plateau, China: these were an open land environment and an understory grassland to represent contrasting light conditions. Both sites are located in an altitude range of 3663–3740 m above sea level (m.a.s.l). This region experiences a typical continental plateau climate, with a short and mild summer and a long, cold winter. The plant growth period lasts approximately 120–140 days. The mean annual temperature and average annual precipitation are −4.18 °C and 425 mm, respectively. Approximately 70–80% of the annual precipitation occurs in July, August, and September. Compared with the understory grassland, *P. griffithii* individuals growing on the open land were exposed to direct sunlight, and the soil conditions were poor. These two sites were chosen because they had similar aspects and topography conditions and the vegetation at these sites was prolific. 

### 4.3. Measurements

In September 2022, a total of 297 individual plants were collected from two habitats, comprising 171 individuals from the open land and 126 from the understory grassland. The selection of individuals was based on the following criteria: (1) only well-developed, obviously healthy, “representative” individuals were selected (but were not necessarily of equal size). (2) Individuals were selected that were some distance away from conspecific neighbors.

**Figure 5:**
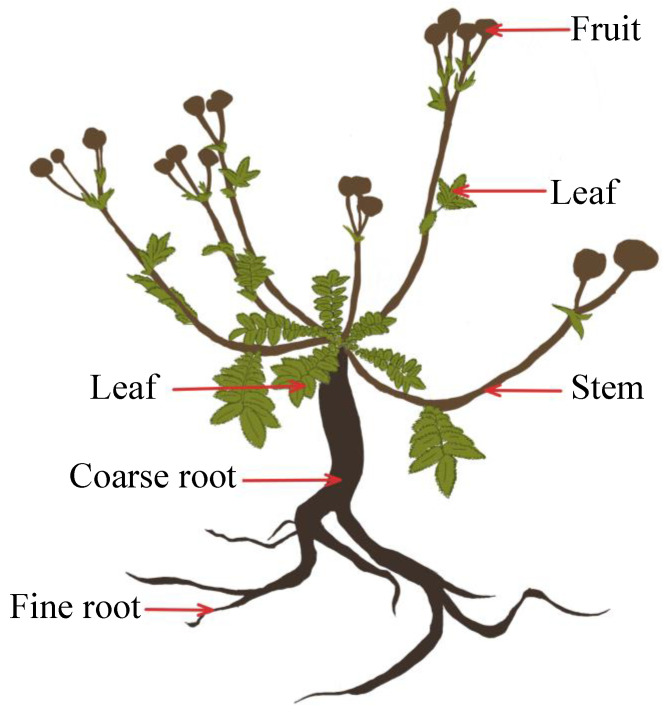
Diagram of a *Potentilla griffithii*, showing the plant organs and other aspects discussed in the text.

Each individual was removed from its microenvironment, in strict accordance with the sampling criteria, and maintained a certain distance from the symbiotic species. The root localization and branching patterns were used to determine the roots of individual plants, and the roots were fully excavated with their soil to minimize the loss of fine roots. A small amount of fine root loss was difficult to avoid, but the loss did not fundamentally affect the biomass data. Fresh plant samples were rinsed and divided into the leaves, aggregate fruits, flower stems, coarse roots, and fine roots (i.e., living roots of <2 mm) (Figure 5).

### 4.4. Trait Measurements

Plant traits (Table 2) were measured for each individual in September 2022. The individual plant was dug out of the soil and brought to the laboratory, washed with distilled water, and air-dried to remove moisture from the surface. The age of each individual was determined by counting the number of leaf scar layers, one by one, starting from the current year leaf. The leaves, stems, fruits, and roots were separated, weighed for fresh mass, and then dried at 55 °C for at least 72 h before the dry mass was measured. Total leaf number (TLN), total stem number (TSN), and total fruit number (TFN) in each individual were recorded. For each individual, all leaves, stems, and roots were scanned, and the total leaf area (TLA), total stem length (TSL), and total root length (TRL), respectively, of each individual was determined using Fiji software (www.fiji.sc, ImageJ) (accessed on 15 September 2022). Stem diameter (StemD) and root diameter (RootD) were determined using a vernier caliper.

After drying, each part of the individual was weighed with an electronic balance (accurate to 0.0001 g) to calculate the traits that correspond to biomass allocation [56]. Total plant dry mass (TDM) was calculated as the sum of the leaf, stem, fruit, and root. The sum of coarse root mass and fine root dry mass was identified as below-ground dry mass (BEDM), and the sum of flower stem, fruit, and leaf dry mass was regarded as above-ground dry mass (ABDM). The plant organ mass fraction was calculated as the ratio of plant organ dry mass to total dry mass. According to the above information, the following traits were calculated:

- Leaf mass per area (LMA) (g cm^−2^): LMA = leaf dry mass (g)/leaf area (cm^2^);
- Mean fruit weight (FruitW) (mg): FruitW = total fruit dry mass (g)/fruit number;
- Specific root length (SRL) (cm g^−1^): SRL = root length (cm)/below-ground dry mass (g);
- Leaf mass fraction (LMF) (g g^−1^): LMF = leaf dry mass (g)/total plant dry mass (g);
- Stem mass fraction (SMF) (g g^−1^): SMF = stem dry mass (g)/total plant dry mass (g);
- Fine root mass fraction (FRMF) (g g^−1^): FRMF = fine root dry mass (g)/total plant dry mass (g);
- Coarse root mass fraction (FRMF) (g g^−1^): FRMF = coarse root dry mass (g)/total plant dry mass (g);
- Below-ground biomass mass fraction (FRMF) (g g^−1^): BEMF = below-ground dry mass (g)/total plant dry mass (g);
- Above-ground biomass mass fraction (ABMF) (g g^−1^): ABMF = above-ground dry mass (g)/total plant dry mass (g);
- Root/shoot ratio (R/S ratio) (g g^−1^): R/S ratio = below-ground dry mass (g)/above-ground dry mass (g).

**Table 2:** List of the plant traits measured in the field experiment.

Cate.	No.	Trait Code	Full Name	Explanations (Unit)
Biomass and allocation	1	TDM	Total dry mass	The total dry mass below and above the ground (g)
	2	ABDM	Above-ground dry mass	The total dry mass of leaves and stems (g)
	3	BEDM	Below-ground dry mass	The total dry mass of roots (g)
	4	R/S	Root-shoot ratio	The ratio of the below-ground and above-ground dry mass
	5	ABMF	Above-ground mass fraction	Above-ground dry mass/total plant dry mass (g g^−1^)
	6	BEMF	Below-ground biomass mass fraction	Root dry mass/total plant dry mass (g g^−1^)
	7	LMF	Leaf mass fraction	Leaf dry mass/total plant dry mass (g g^−1^)
	8	SMF	Stem mass fraction	Stem dry mass/total plant dry mass (g g^−1^)
	9	FRMF	Fine root mass fraction	Fine root dry mass/total plant dry mass (g g^−1^)
	10	CRMF	Coarse root mass fraction	Coarse root dry mass/total plant dry mass (g g^−1^)
The whole-plant morphology	11	TLN	Total leaf number	Total leaves per individual (No.)
	12	TFN	Total fruit number	Total aggregate fruits per individual (No.)
	13	TSN	Total stem number	Total stems per individual (No.)
	14	TLA	Total leaf area	The sum of all the leaf area (cm^2^)
	15	StemD	Stem diameter	Mean stem diameter of two dimensions per stem (mm)
	16	StemL	Total stem length	Mean stem length per plant (cm)
	17	RootD	Root diameter	Mean coarse root diameter of two dimensions (mm)
	18	TRL	Root length	The sum of all the root lengths (cm)
	19	FruitW	Mean fruit weight	Mean aggregate fruit weight (mg)
	20	SRL	Specific root length	Root length per mass (cm/g)
	21	LMA	Leaf mass per area	Leaf mass per unit leaf area (g/cm^2^)

### 4.5. Statistical Analysis

Differences between the plant populations of the two distinct habitats were tested using Student’s *t*-test after the homogeneity of variance was analyzed via an F-test (*p* < 0.05). For the comparison of each plant trait, a log10 transformation was applied to normalize the data set. Data were log10-transformed to determine the relationships between plant morphological traits, organ mass fraction, and plant age by using linear regression analysis. Principal component analysis (PCA) was conducted to test the relationships among plant traits, using the “FactoMineR” package in R [71].

## 5. Conclusions

In the present study, we have demonstrated the feasibility of determining the age of *P. griffithii* perennial grass specimens by counting the leaf scars. Through this method, we determined the age structure and growth strategy of *P. griffithii* populations in two typical habitats in the western Sichuan Plateau. In the open land environment, the age structure is relatively younger than in the understory, and *P. griffithii* plants tend to allocate more photosynthate terminal organs (leaf and fine root organs) to absorb more resources, with the reproductive organs (flower stem and aggregate fruits) to expand the population. The *P. griffithii* population in understory habitats is in the middle-age stage and concentrates more photosynthate in the coarse root part to support the plant. In addition, we found that in the open field, the age of potentilla plants offers a good correlation with various traits, meaning that the plants can be used as a good predictor of plant growth. Our findings advance the scientific knowledge of *P. griffithii* growth in the plateau and provide scientific support for large-scale breeding.

## Figures and Tables

**Table 1:** Comparison of plant biomass accumulation, biomass allocation, and morphological trains between two habitats in Gaoersi Mount.

Cate.	Traits	Open Land		Understory Grassland		*p*-Value
		No. of Samplings	Mean + SE	No. of Samplings	Mean + SE	
Biomass and allocation	TDM (g)	171	3.21 ± 0.27	126	2.93 ± 0.27	NS
	ABDM (g)	171	1.13 ± 0.11	124	0.62 ± 0.065	<0.001
	BEDM (g)	171	2.07 ± 0.17	126	2.31 ± 0.21	NS
	R/S	171	2.11 ± 0.097	124	4.41 ± 0.29	<0.001
	ABMF (g g^−1^)	171	0.36 ± 0.008	124	0.22 ± 0.0076	<0.001
	BEMF (g g^−1^)	171	0.64 ± 0.008	126	0.78 ± 0.0076	<0.001
	LMF (g g^−1^)	171	0.31 ± 0.0077	126	0.21 ± 0.0074	<0.001
	SMF (g g^−1^)	90	0.077 ± 0.0056	37	0.059 ± 0.0074	NS
	FRMF (g g^−1^)	165	0.26 ± 0.014	124	0.21 ± 0.017	<0.01
	CRMF (g g^−1^)	171	0.55 ± 0.0094	126	0.69 ± 0.0095	<0.001
The whole-plant morphology	TLN (No.)	170	21.73 ± 2.13	124	11.93 ± 1.29	<0.001
	TFM (No.)	90	19.47 ± 2.35	37	14.99 ± 3.44	NS
	TSN (No.)	90	2.98 ± 0.27	39	2.01 ± 0.31	<0.001
	TLA (cm^2^)	122	73.34 ± 4.53	126	64.77 ± 3.74	NS
	StemD (mm)	91	2.24 ± 0.062	39	2.11 ± 0.091	NS
	StemL (cm)	91	17.65 ± 0.78	37	22.74 ± 0.79	<0.001
	RootD (mm)	171	7.55 ± 0.26	126	8.49 ± 0.27	<0.01
	TRL (cm)	171	25.18 ± 1.28	126	24.41 ± 0.95	NS
	FruitW (mg)	90	10.72 ± 0.91	37	8.1 ± 0.32	<0.05
	SRL (cm g^−1^)	171	21.98 ± 1.61	125	20.41 ± 2.77	NS
	LMA (mg cm^−2^)	121	10.89 ± 0.44	126	7.65 ± 0.14	<0.001

Plant traits are log10-transformed to meet the requirements (normality and homogeneity of variance) for data analysis. Full names of the plant traits are given in Table 2.

## Data Availability

Not applicable.

## Supplementary Materials

The following supporting information can be downloaded at: https://www.mdpi.com/article/10.3390/plants12162920/s1, Figure S1: Pictures of common garden, open land, understory grassland habitats; Table S1: Relationship, coefficient of determination (R2) and *p* value among leaf traits in Figure 3 and Figure 4. Shaded cells were used to improve the table readability; Table S2: PCA of plant age, biomass accumulation, biomass allocation, and the whole plant morphological traits.
