# Supplementary material for: Age Determination and Growth Characteristics of the Potentilla griffithii: A Comparison of Two Different Habitats in Western Sichuan Plateau, China
Source: Plants (Basel). 2023 Aug 11;12(16):2920. doi: 10.3390/plants12162920 (PMC10459867; doi:10.3390/plants12162920)
Supplement: Supplementary file 1 [file plants-12-02920-s001.zip › plants-2525536-supplementary.pdf]

Supplemental Materials

Table S1 Relationship, coefficient of determination (R^2) and *P* value among leaf traits in **Figure 3 and Figure 4**. Shaded cells were used to improve the table readability.

Habitat	X	Y	Relationship	R^2	P
Open land	Log10 Age	Log10FRMF	Y = (-0.88294) + 0.3683*x	0.16	<0.001
		Log10CRMF	Y = (-0.37131) + 0.1851*x	0.13	<0.001
		Log10BEMF	n.s.	-	-
		Log10LMF	Y = (-0.39676) + (-0.24079) *x	0.15	<0.001
		Log10SMF	Y = (-1.6862) + 0.70315*x	0.17	<0.001
		Log10ABMF	n.s.	-	-
		Log10 R/S	n.s.	-	-
		Log10BEDM	Y = (-0.58717) + 1.3244*x	0.51	<0.001
		Log10ABDM	Y = (-0.81228) + 1.2386*x	0.49	<0.001
		Log10TDM	Y = (-0.37439) + 1.2972*x	0.54	<0.001
		Log10LMA	n.s.	-	-
		Log10FruitW	n.s.	-	-
		Log10SRL	Y = 1.6578 + (-0.83414) *x	0.4	<0.001
		Log10TLN	Y = 0.62767 + 0.99637*x	0.42	<0.001
		Log10TFN	Y = 0.19439 + 1.335*x	0.33	<0.001
		Log10TSN	Y = (-0.13214) + 0.76045*x	0.26	<0.001
		Log10TLA	Y = 1.3897 + 0.75623*x	0.26	<0.001
		Log10TRL	Y = 1.0781 + 0.48689*x	0.086	<0.01
		Log10RootD	Y = 0.5208 + 0.59362*x	0.48	<0.001
		Log10StemD	Y = 0.20977 + 0.18916*x	0.27	<0.001
		Log10StemL	Y = 0.94362 + 0.40317*x	0.17	<0.001
Understory grassland	Log10 Age	Log10FRMF	n.s.	-	-
		Log10CRMF	n.s.	-	-
		Log10BEMF	n.s.	-	-
		Log10LMF	n.s.	-	-
		Log10SMF	n.s.	-	-
		Log10ABMF	n.s.	-	-
		Log10 R/S	n.s.	-	-
		Log10BEDM	Y = 0.52019 + (-0.50204) *x	0.047	<0.05
		Log10ABDM	Y = (-0.34555) + (-0.062629) *x	0.0014	<0.05
		Log10TDM	Y = 0.61443 + (-0.47825) *x	0.047	<0.05
		Log10LMA	n.s.	-	-
		Log10FruitW	n.s.	-	-
		Log10SRL	Y = 1.643 + (-0.73089) *x	0.19	<0.001
		Log10TLN	n.s.	-	-
		Log10TFN	n.s.	-	-
		Log10TSN	Y = 0.53587 + (-0.52884) *x	0.11	<0.05

	Log10TLA	Y = 1.4669 + 0.37904*x	0.053	<0.05
	Log10TRL	Y = 1.1134 + 0.34806*x	0.12	<0.001
	Log10RootD	Y = 0.57316 + 0.49234*x	0.33	<0.001
	Log10StemD	n.s.	-	-
	Log10StemL	n.s.	-	-

- Indicates no item.

Table S2 PCA of plant age, biomass accumulation, biomass allocation, and the whole plant morphological traits.

Site		Plant Traits	PC1	PC2	PC3
Open land	Age	Age	0.83	-0.1	0.11
	Biomass & allocation	FRMF (g g^{-1})	-0.017	-0.17	0.28
		CRMF (g g^{-1})	0.31	-0.79	-0.13
		BEMF (g g-1)	-0.0038	-0.97	0.024
		LMF (g g-1)	-0.41	0.84	-0.012
		SMF (g g-1)	0.58	0.66	-0.0097
		ABMF (g g-1)	0.0038	0.97	-0.024
		R/S	0.0201	-0.83	0.11
		BEDM (g)	0.93	-0.17	0.032
		ABDM (g)	0.93	0.23	0.034
		TDM (g)	0.96	-0.021	0.034
	The whole-plant morphology	LMA (mg cm^{-2})	-0.054	0.63	0.14
		FruitW (mg)	0.097	-0.048	-0.62
		SRL (cm g^{-1})	-0.52	0.31	0.22
		TLN (No.)	0.87	0.15	0.33
		TFM (No.)	0.92	0.21	0.19
		TSN (No.)	0.81	0.13	0.39
		TLA (cm^2)	0.94	0.099	-0.13
		TRL (cm)	0.64	-0.31	0.14
		RootD (mm)	0.88	-0.21	-0.12
		StemD (mm)	0.64	0.19	-0.53
		StemL (cm)	0.72	0.25	-0.37
Understory grassland	Age	Age	-0.15	0.23	0.66
	Biomass & allocation	FRMF (g g^{-1})	-0.19	0.18	-0.15
		CRMF (g g^{-1})	0.16	-0.84	0.23
		BEMF (g g-1)	-0.043	-0.95	0.21
		LMF (g g-1)	-0.24	0.89	-0.19
		SMF (g g-1)	0.39	0.82	-0.017
		ABMF (g g-1)	0.044	0.95	-0.18
		R/S	-0.11	-0.74	0.11
		BEDM (g)	0.91	-0.26	-0.18
		ABDM (g)	0.96	0.062	-0.18
		TDM (g)	0.93	-0.19	-0.18
	The whole-plant morphology	LMA (mg cm^{-2})	0.17	0.079	0.36
		FruitW (mg)	0.052	0.55	0.028
		SRL (cm g^{-1})	-0.11	-0.28	-0.52
		TLN (No.)	0.92	0.031	-0.041
		TFM (No.)	0.98	0.0031	-0.019

	TSN (No.)	0.96	-0.094	-0.031
	TLA (cm^2)	0.68	0.12	0.47
	TRL (cm)	0.18	0.21	0.71
	RootD (mm)	0.068	0.26	0.88
	StemD (mm)	0.59	-0.32	-0.011
	StemL (cm)	0.64	0.52	0.0096

Shaded cells were used to improve the table readability. See Table 1 for abbreviations of plant traits.

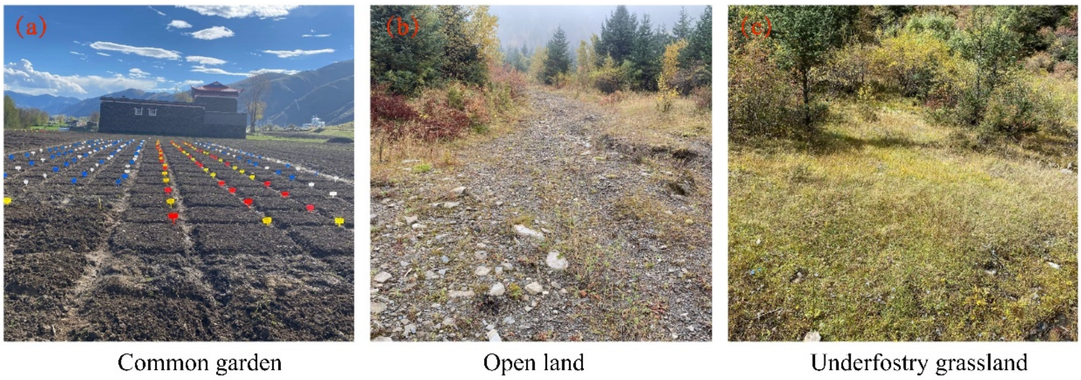

Figure S1. Pictures of common garden, open land, understory grassland habitats
